# SSF of steam-pretreated wheat straw with the addition of saccharified or fermented wheat meal in integrated bioethanol production

**DOI:** 10.1186/1754-6834-6-169

**Published:** 2013-11-29

**Authors:** Borbála Erdei, Dóra Hancz, Mats Galbe, Guido Zacchi

**Affiliations:** 1 Department of Chemical Engineering, Lund University, P.O. Box 124, SE-221 00 Lund, Sweden; 2 Department of Experimental Medical Science, Lund University, BMC D14, 221 84 Lund, Sweden

**Keywords:** SSF, SSCF, Wheat meal hydrolysate, Fermented wheat meal, Steam-pretreated wheat straw, Glucose and xylose co-fermentation

## Abstract

**Background:**

Integration of second-generation (2G) bioethanol production with existing first-generation (1G) production may facilitate commercial production of ethanol from cellulosic material. Since 2G hydrolysates have a low sugar concentration and 1G streams often have to be diluted prior to fermentation, mixing of streams is beneficial. Improved ethanol concentrations in the 2G production process lowers energy demand in distillation, improves overall energy efficiency and thus lower production cost. There is also a potential to reach higher ethanol yields, which is required in economically feasible ethanol production. Integrated process scenarios with addition of saccharified wheat meal (SWM) or fermented wheat meal (FWM) were investigated in simultaneous saccharification and (co-)fermentation (SSF or SSCF) of steam-pretreated wheat straw, while the possibility of recovering the valuable protein-rich fibre residue from the wheat was also studied.

**Results:**

The addition of SWM to SSF of steam-pretreated wheat straw, using commercially used dried baker’s yeast, *S. cerevisiae*, resulted in ethanol concentrations of about 60 g/L, equivalent to ethanol yields of about 90% of the theoretical. The addition of FWM in batch mode SSF was toxic to baker’s yeast, due to the ethanol content of FWM, resulting in a very low yield and high accumulation of glucose. The addition of FWM in fed-batch mode still caused a slight accumulation of glucose, but the ethanol concentration was fairly high, 51.2 g/L, corresponding to an ethanol yield of 90%, based on the amount of glucose added.

In batch mode of SSCF using the xylose-fermenting, genetically modified *S. cerevisiae* strain KE6-12, no improvement was observed in ethanol yield or concentration, compared with baker’s yeast, despite the increased xylose utilization, probably due to the considerable increase in glycerol production. A slight increase in xylose consumption was seen when glucose from SWM was fed at a low feed rate, after 48 hours, compared with batch SSCF. However, the ethanol yield and concentration remained in the same range as in batch mode.

**Conclusion:**

Ethanol concentrations of about 6% (w/v) were obtained, which will result in a significant reduction in the cost of downstream processing, compared with SSF of the lignocellulosic substrate alone. As an additional benefit, it is also possible to recover the protein-rich residue from the SWM in the process configurations presented, providing a valuable co-product.

## Background

The production of bioethanol from sugar- and starch-based raw materials such as sugar cane in Brazil and maize in the US, referred to as first-generation (1G) production, is well established today. However, the sustainability of this technique has been questioned as it makes use of materials otherwise used for food [1,2]. Considerable effort has thus been devoted to the development of technologies for biofuel production from lignocellulosic biomass. Despite extensive research over the past thirty years, so-called second-generation (2G) bioethanol production is still not economically feasible. Although commercial production has started, the expected expansion of 2G ethanol production has not been realized.

Due to the lack of experience of large-scale production, the estimated cost of 2G bioethanol varies considerably [2,3]. Besides the capital cost of the plant, the main parameters influencing the production cost of ethanol from lignocellulosic materials are the cost of feedstock, enzyme, and energy. High ethanol yield and concentration are also necessary to reduce production costs [3,4]. Higher ethanol concentrations can be achieved by increasing the amount of water-insoluble solids (WIS), however, this usually results in a decrease in yield due to inhibition caused by degradation products, or reduced mass transfer [5,6].

Integration of existing 1G bioethanol production with 2G ethanol production may facilitate the introduction of cellulosic material in bioethanol production. The ethanol concentration can be increased by the addition of the starch-derived hydrolysate from the 1G process. We have shown in a previous study that the addition of pre-saccharified wheat meal to the simultaneous saccharification and fermentation (SSF) of steam-pretreated wheat straw (SPWS) not only increased the ethanol concentration, but also the ethanol yield, compared with the stand-alone configurations [7]. However, the configuration used in our previous study did not allow utilization of the protein-rich material (distiller’s dried grains with solubles) which can be used as animal feed. Tang et al. later demonstrated that the addition of corn hydrolysate not only increased the ethanol concentration, but could also provide a source of organic nutrients (source of nitrogen) in SSF of lignocellulosic residue [8].

Agricultural residues, such as wheat straw, contain significant amounts of hemicellulose, which makes xylose fermentation an important part of the process. The yeast, *S. cerevisiae* is a robust, widely used industrial microorganism, but it is not able to ferment xylose. Xylose-fermenting pathways have therefore been introduced into *S. cerevisiae*[9]. The strain TMB3400 [10] carries the XYL1 and XYL2 genes of *P. stipitis*, which encode for xylose reductase (XR) and xylitol dehydrogenase (XDH) [11,12]. KE6-12 is a mutant strain developed from TMB3400 by a combination of different evolutionary engineering strategies and random mutagenesis (Albers *et al.*: Evolutionary engineering for development of improved xylose utilization capacity and inhibitor tolerance in an industrial Saccharomyces cerevisiae strain, manuscript in preparation), which has demonstrated an improved ability to utilize xylose [13], especially in the fed-batch addition of glucose-containing material [14].

In the present study, the supernatant from saccharified wheat meal (SWM) or from fermented wheat meal (FWM) was added to SSF of SPWS using baker’s yeast, *S. cerevisiae* to assess the effect on ethanol concentration and yield. Four different process configurations were employed in an attempt to integrate 1G and 2G bioethanol production. The modified strain KE6-12 was also used in simultaneous saccharification and co-fermentation (SSCF) with batch or fed-batch addition of SWM to investigate whether the addition of SWM increased the xylose utilization of this strain.

## Results and discussion

In an attempt to increase the ethanol concentration in the broth, four different process configurations were investigated in the present study; i.e. integration in SSF after steam pretreatment of the lignocellulosic material with saccharified wheat meal (SWM) with different WIS contents or with saccharified and fermented wheat meal (FWM).

### Fermentation of saccharified wheat meal prior to SSF

**Figure 1:**
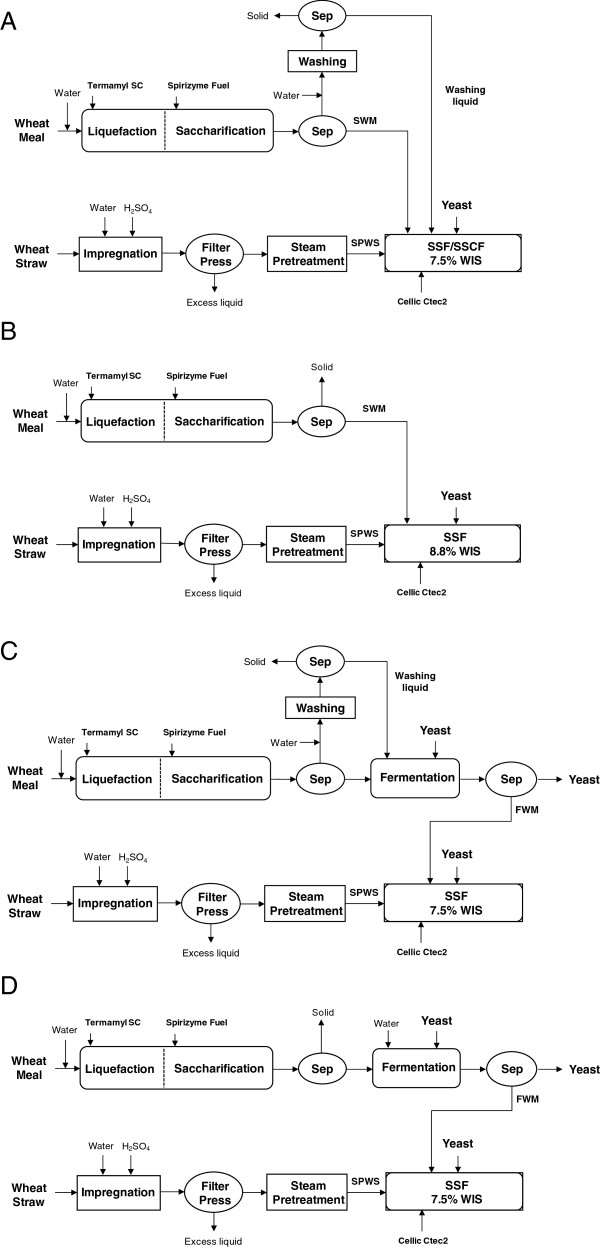
**Flow chart showing the experimental design for the assessment of simultaneous saccharification and (co-)fermentation (SSF/SSCF).** SSF/SSCF of steam-pretreated wheat straw (SPWS) was mixed with: **(A)** a mixture of saccharified wheat meal (SWM) and the washing liquid, **(B)** SWM, or **(C, D)** fermented wheat meal (FWM) at a WIS content of **(A, C, D)** 7.5% (w/w) or **(B)** 8.8% (w/w). In Configuration C SSF was performed in batch mode and in Configuration D in fed-batch mode. Sep: Separation by centrifugation.

**Figure 2:**
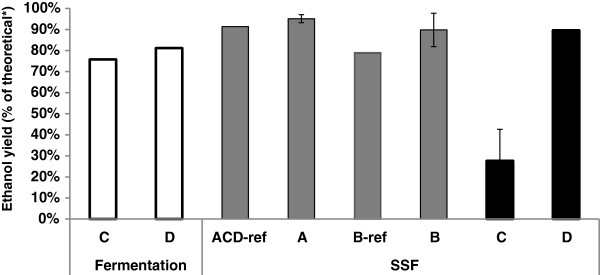
**Ethanol yield from fermentation (fed-batch, no shaded C, D) or SSF.** SSFs were performed in batch mode with the addition of SWM (grey, A: 7.5% (w/w) WIS, B: 8.8% (w/w) WIS) and in batch (C) or fed-batch (D) mode with addition of FWM (black). ACD-ref and B-ref experiments were performed with addition of water instead of SWM or FWM. The various configurations (see Table 1) were performed using baker’s yeast. *Based on the total amount of glucose added to fermentation or SSF. Error bars are based on standard deviation for duplicate experiments.

The saccharified wheat meal was fermented in fed-batch mode to produce FWM, which was then used in SSF in Configurations C and D (Figure 1). Water was used to wash the filter cake of the wheat meal to recover some of the sugars. Approximately 50% of the sugars in the filter cake were recovered, corresponding to a 30% increase in the total amount of glucose added in Configuration C, compared with Configuration D (when the filter cake was not washed). The initial glucose concentration in Configuration C and D was 127.2 and 100.2 g/L, respectively. During the first eight hours of SSF, when the glucose concentration was high, the average ethanol production rate was almost 7 g/L h. However, after 48 hours, when the ethanol concentration reached 91.2 g/L, fermentation ceased resulting in an ethanol yield of 76% of the theoretical (Figure 2), and leaving 21 g/L residual glucose. This ethanol titre is at the high end of the range reported for the tolerance of yeast to ethanol [15]. The sugar remaining in the broth can be fermented in the subsequent step of SSF, thus there is no loss in this process configuration (C). Glycerol was produced at a concentration of 8.5 g/L, corresponding to a yield of 0.032 g/g glucose, which is common in fermentation to produce bioethanol using *Saccharomyces cerevisiae* due to the formation of biomass [16,17].

In an industrial process, the amount of glucose originating from the starch material would be higher than in the laboratory set-up, as sugars can be recovered from the filter cake by thorough rinsing and counter-current extraction [18]. Furthermore, the washing stream could be reused at an earlier stage of the process. The fermentation step must be optimized to achieve a high ethanol concentration and thus a high ethanol yield. However, the present study was concerned with the effect of FWM addition on SSF of wheat straw so no attempt was made to recover starch sugars. Due to the lower amount of sugars added, see Table 1, the fermentation step in Configuration D resulted in a final ethanol concentration of 75.1 g/L, corresponding to an ethanol yield of 81% of the theoretical (Figure 2) due to a reduction in ethanol inhibition.

**Table 1:** Experimental conditions, including the amount of sugars added to SSF/SSCF and fermentation (Ferm.)

**Config.**	**Process step**	**Operation mode**		**Strain**	**WIS SPWS**	**Liquid added**	**Feed start-stop**	**Glucose**								**Xylose**		**EtOH**	
								**SPWS**		**SWM**		**WL**		**FWM**		**SPWS**		**FWM**	
						**At 0 h**		**Solid**	**Liquid**	**At 0 h**	**Feed**	**At 0 h**	**Feed**	**At 0 h**	**Feed**	**Solid**	**Liquid**	**At 0 h**	**Feed**
					% of total w		h	g	g	g	g	g	g	g	g	g	g	g	g
A,C,D	SSF	Batch	Ref	Dry baker’s yeast	7.5	H_2_O	-	48.9	4.4	-	-	-	-	-	-	5.6	20.9	-	-
A	SSF	Batch	a, b		7.5	SWM + WL	-	48.9	4.4	52.1	-	17.3	-	-	-	5.6	20.9	-	-
B	SSF	Batch	Ref		8.8	H_2_O	-	57.4	5.2	-	-	-	-	-	-	6.5	24.5	-	-
B	SSF	Batch	a, b		8.8	SWM	-	57.4	5.2	60.9	-	-	-	-	-	6.5	24.5	-	-
C	Ferm.	Fed-batch			-	WL	SWM 8-32	-	-	-	176.0	57.0	-	-	-	-	-	-	-
C	SSF	Batch	a, b		7.5	FWM	-	48.9	4.4	-	-	-	-	7.1	-	5.6	20.9	30.9	-
D	Ferm.	Fed-batch			-	SWM + H_2_O	SWM 8-32	-	-	50.1	129.1	-	-	-	-	-	-	-	-
D	SSF	Fed-batch			7.5	-	FWM 24-96	48.9	4.4	-	-	-	-	-	0.0	5.6	20.9	-	25.5
A	SSCF	Batch		KE6-12	7.5	SWM	-	48.9	4.4	52.1	-	17.3	-	-	-	5.6	20.9	-	-
A	SSCF	Fed-batch	I		7.5	-	SWM 48-96	48.9	4.4	-	52.1	-	17.3	-	-	5.6	20.9	-	-
A	SSCF	Fed-batch	II		7.5	-	SWM 24-96	48.9	4.4	-	52.1	-	17.3	-	-	5.6	20.9	-	-

The various configurations (Config.) are described in the text.

WIS: water-insoluble solids, SPWS: steam-pretreated wheat straw, SWM: saccharified wheat meal, FWM: fermented wheat meal, WL: washing liquid, EtOH: ethanol. Ref: reference experiments were performed with addition of water.

a, b: experiments performed in duplicate.

### Simultaneous saccharification and fermentation

SSF experiments were performed with the addition of SWM (Configurations A and B, see Figure 1A and Figure 1B) or the addition of FWM (Configurations C and D, see Figure 1C and Figure 1D). Reference experiments were performed with the addition of water for comparison of yields and concentrations. Table 2 presents the experimental results and calculated yields for the SSF experiments based on the total amount of glucose and glucan added to SSF.

**Table 2:** Substrate, product concentrations and yields obtained after 120 hours of SSF

**Config.**	**Glu**^**1**^	**EtOH**^**1**^	**Glycerol**^**1**^	**Y**_**glycerol**_	**EtOH prod. rate**^**2**^	**Y**_**EtOH**_	
	**g/L**	**g/L**	**g/L**	**g/g glu**	**g/L h**	**%**	**g/g glu**
A,C,D^Ref^	0.0	24.9	1.9	0.034	0.75	91	0.47
A^a^	3.2	60.3	3.9	0.030	0.88	96	0.49
A^b^	3.9	58.7	3.7	0.029	1.07	94	0.48
B^Ref^	0.0	26.3	1.2	0.018	0.26	79	0.40
B^a^	4.9	56.1	8.5	0.065	0.50	84	0.43
B^b^	3.8	63.2	2.7	0.021	0.68	95	0.49
C^a^	43.5	37.4 (3.8^3^)	3.3 (0.0^3^)	0.000	0.48	16	0.08
C^b^	23.8	43.9 (10.3^3^)	4.4 (0.4^3^)	0.006	0.26	37	0.19
D	10.5	51.2 (25.6^3^)	7.9 (3.1^3^)	0.057	1.59	90	0.46

The experimental configurations (Config.) performed with baker’s yeast are described in Table 1.

Glu: glucose, EtOH: ethanol; Y: yield, ^Ref^: reference experiment.

^a, b^: experiments performed in duplicate.

^1^ Concentration at the end of the SSF experiment.

^2^ Calculated from data for the first eight hours.

^3^ Produced during SSF.

#### SSF with SWM

**Figure 3:**
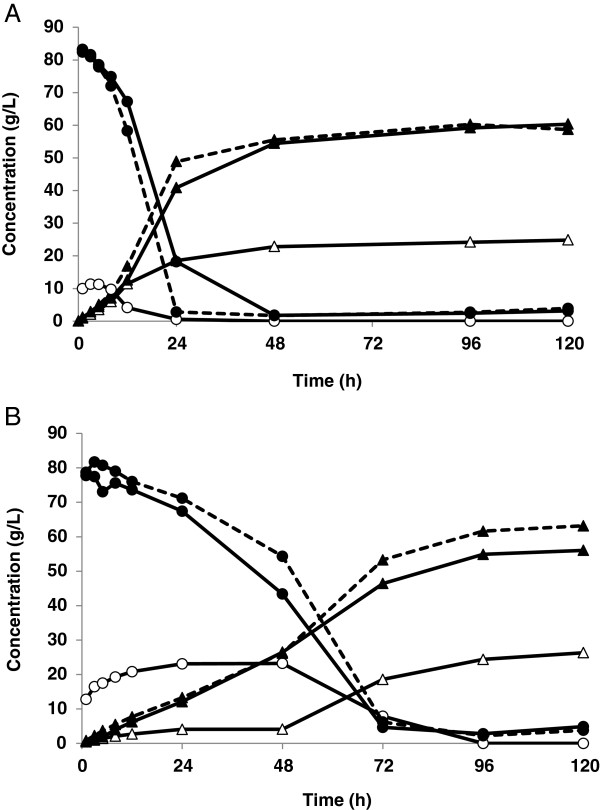
**Glucose (circles) and ethanol (triangles) concentration during SSF.** SSF had 7.5% (w/w) WIS, in Configuration A **(A)** and 8.8% (w/w) WIS in Configuration B **(B)**. Empty symbols show data from reference experiments with addition of water instead of SWM. Experiments with SWM (filled symbols) were performed in duplicate (solid and dashed lines).

Figure 3 shows the concentrations of glucose and ethanol versus time during SSF with 7.5 wt-% (Figure 3A) or 8.8 wt-% (Figure 3B) WIS, without and with the addition of SWM. A final average ethanol concentration of about 60 g/L was reached when SWM was added to SSF with 7.5 wt-% WIS, which is more than double that in the reference experiment (24.9 g/L). A slight increase in the ethanol yield was observed with SWM addition, which is probably due to the greater proportion of readily available glucose from SWM that does not need to be hydrolysed in the SSF step (see Table 2). Because of the higher initial sugar concentration, the ethanol production rate increased to an average of about 0.95 g/L/h, compared with 0.75 g/L/h without the addition of SWM.

The higher WIS concentration, 8.8 wt-%, resulted in a lower ethanol yield (Table 2), only 79% of theoretical, as a result of the increased inhibitor concentration (2.1 g/L furfural versus 1.6 g/L in SSF with 7.5 wt-% WIS at time 0 hour). Therefore, there was only little increase in the ethanol concentration in the reference experiment of 8.8% WIS (26.3 g/L) compared with the reference of 7.5 wt-% WIS (24.9 g/L). A considerable decrease was observed in the mean ethanol production rate during the first 8 hours (from 0.75 to 0.26 g/L/h) due to a longer lag phase. It took 24 hours to assimilate furfural in SSF with 8.8 wt-% WIS, while only 5 hours was needed with 7.5 wt-% WIS. The toxic environment in the slurry after steam pretreatment of the wheat straw is inhibitory to both the yeast [19,20] and the enzymes [21,22]. Öhgren et al. observed similar behaviour with increased WIS concentration [23]. However, the tolerance of *S. cerevisiae* to inhibitors can be improved by cultivating the yeast on hydrolysate from lignocellulose. Alkasrawi et al. reported a significant improvement in performance during SSF, leading to increased ethanol productivity [24].

In the present study, it was found that the addition of SWM enhanced ethanol productivity during the first eight hours. The average ethanol production rate increased from 0.26 to about 0.6 g/L/h (Table 2) during this period in Configuration B (Figure 1B). This may be due to the high initial glucose concentration, as the concentration of inhibitors was identical in the integrated and in the reference experiments, or to the positive effect of the extra nutritional value of SWM. The addition of similar starch-based materials has been shown to enhance SSF in a previous study on SPWS using partly saccharified wheat meal [7], and a study on lignocellulosic residues from furfural production together with hydrolysed corn kernels [8]. In a recent study, grain mash was used as the sole nutrient source for the preparation of an inoculum for SSF with high substrate loading [25]. The inoculum was found to be sufficiently robust to yield high ethanol concentrations without the addition of extra nutrients.

More glycerol was produced by the same amount of yeast in SSF when SWM was added (Table 2). Glycerol is produced by *S. cerevisiae* in response to osmotic stress, which is created in media containing hyperosmolar glucose concentrations [26]. Intracellular glycerol concentration is produced by the high-osmolarity glycerol pathway [27], and is essential for the growth of the cell, enabling enzymes to function under conditions of reduced water activity [17,26]. However, ethanol also causes a reduction in water activity, and it has been shown that there is an increase in glycerol production in yeast cells resulting from this stress [15]. Glycerol diffuses through the cell membrane, requiring the continuous synthesis of glycerol to maintain intracellular protection.

The higher sugar concentration resulting from the addition of SWM leads to an ethanol concentration of about 60 g/L, which is more than double that without SWM (26.3 g/L) (see Figure 3B). The ethanol yields achieved in SSF, with SWM addition, were between 84 and 95% of the theoretical. It would be of interest to further increase the WIS, but in the current laboratory configuration the maximum WIS obtainable using SPWS (with 11.7% WIS) and the same amount of wheat meal and wheat straw was 8.8%. However, higher values could be obtained in a large-scale process, since continuous steam pretreatment often provides pretreated slurries with WIS above 15% [14], or even 30% [28].

One advantage of the configurations with SWM addition is that the solid residues remaining after washing the filter cake is a good source of protein that can be sold as a co-product on the animal feed market [29], as it is not contaminated by any compounds from the lignin residue of the 2G ethanol production, thus improving the economics of the process. The mixture of this material with yeast residues after fermentation is sold as distiller’s dried grains with solubles (DDGS), and is the major co-product resulting from bioethanol production from maize and wheat in today’s 1G ethanol plants [30,31]. At the same time, the lignin residues from the 2G plant can be burnt to produce heat and electricity. The current configuration is advantageous compared to that used in a previous study, where the pre-saccharified wheat meal (containing the solid residue) was added to SSF of SPWS [7]. In the previous configuration, the protein-rich solids were mixed with the lignin residue, and could thus probably only be used to produce heat and power.

#### SSF with FWM

**Figure 4:**
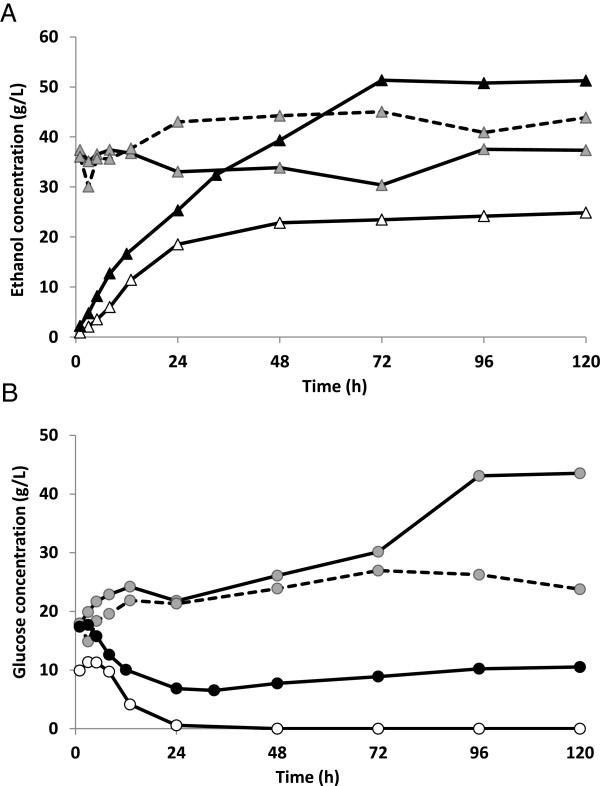
**Ethanol (A) and glucose (B) concentrations during SSF of SPWS (7.5% (w/w) WIS).** SSF was performed with addition of water (reference- empty symbols), and with addition of FWM (batch addition Configuration C: shaded symbols and fed-batch Configuration D: filled symbols). Batch experiments were performed in duplicate, as indicated by the solid (Configuration C_a_) and dashed (Configuration C_b_) lines.

One way of integrating the ethanol production processes from wheat straw and wheat meal is to use already fermented wheal meal for dilution in SSF, in order to increase the ethanol concentration in the broth after fermentation, which in turn would decrease the energy needed in downstream processing to recover the ethanol [32]. SSF was initially carried out with 7.5 wt-% WIS (as in Configuration C) with batch addition of FWM. Figure 2 and Figure 4 show the ethanol yields achieved, and the ethanol and glucose concentrations measured during SSF with FWM addition, respectively.

The initial ethanol concentration (33.6 g/L) increased to only around 40 g/L, resulting in a very low ethanol yield in batch SSF, less than 40% of the theoretical, respectively (see Table 2), while great amount of glucose accumulated. These results indicate strong inhibition, which is most likely to be induced by the ethanol added with the FWM to SSF in batch mode. Control experiments (results not shown) with the same amount of ethanol added to SSF have shown that the whole FWM broth was not more inhibitory than only ethanol added at the same concentration. This proves that a high concentration of added ethanol has an effect on the microbial activity. However, the ethanol concentration reported to have effects on metabolism [33] or to cause complete inhibition [34] of the growth of *S. cerevisiae* are also significantly higher than the initial concentration in the experiments performed in this study.

Metabolic activity of the yeast may also be more affected, at the initial stage of rehydration, when dry yeast is used as fermentation organism. Metabolic activity must be regained, which might be difficult in an environment with the presence of several inhibitory compounds and high total solid loading. These circumstances may have also lead to unstable behaviour of the yeast, i.e. difference in ethanol production between the replicates and glucose accumulation of SSF with batch addition of FWM (see Figure 4). The decline in glucose concentration in Configuration C^b^ is a result of contamination of lactic acid bacteria, which produced 8.9 g/L lactic acid during the last 48 hours. Thus, it is likely that the final glucose concentration in Configuration C^b^ would have been similar as in Configuration C^a^.

To avoid ethanol toxicity in the critical first hours of SSF, a delay in the addition of FWM and a slow addition rate in fed-batch mode was considered. Therefore, in the fed-batch experiment of Configuration D (Figure 1D) feeding of FWM was fed starting after 24 hours allowing the yeast to adapt to the inhibitory environment. Fermentation started with a high productivity of 1.59 g/L/h (see Table 2). Most of the glucose was converted to ethanol during the first 24 hours, before FWM was added. Despite feeding in a later phase of SSF, addition of FWM, containing ethanol caused immediate inhibition, and no more glucose was fermented under the fed-batch phase. This resulted in the accumulation of the glucose released by the hydrolysis of the SPWS, with a final concentration in SSF of 10.5 g/L. Therefore, the increase in ethanol concentration, seen in Figure 4A, must be due to the addition of ethanol with the FWM. Although an ethanol concentration as high as 50 g/L could be reached with fed-batch addition, cell death may have already occurred before the addition of FWM or the severe toxicity of the added ethanol may have caused fermentation to cease.

#### SSCF with SWM feed using the xylose-fermenting yeast

**Figure 5:**
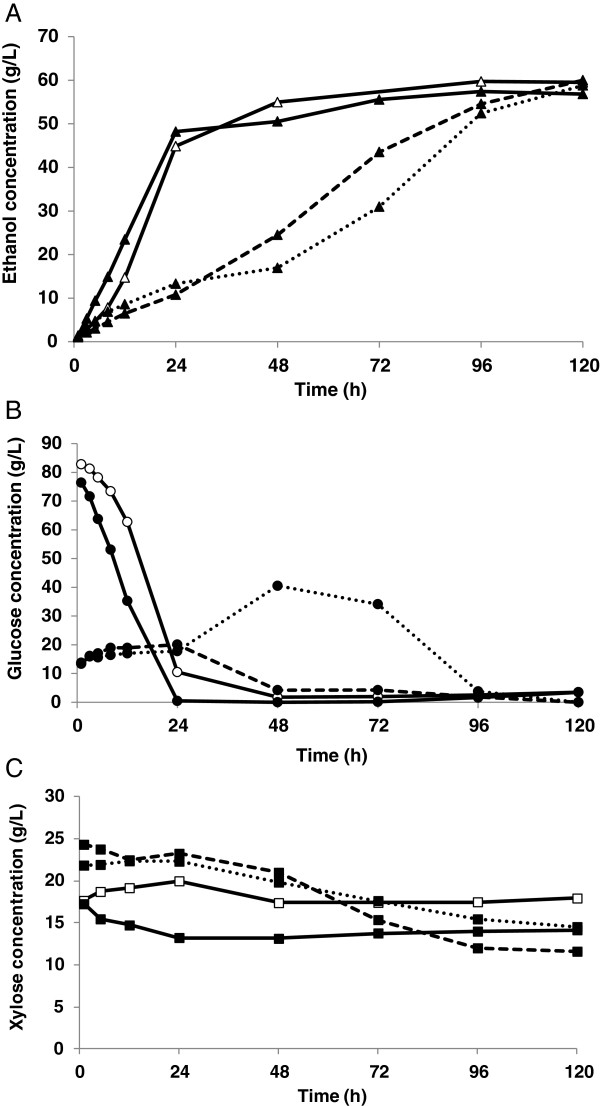
**Ethanol (A), glucose (B) and xylose (C) concentrations during SSCF.** Empty and filled symbols represent SSCF of SPWS (7.5% (w/w) WIS in Configuration A, see Figure 1) using dried baker’s yeast and KE6-12, respectively. Batch experiments: solid lines, fed-batch 48–96 hours: dashed lines and fed-batch 24–96 hours: dotted lines. Data obtained using baker’s yeast is the mean value of duplicate experiments.

Figure 5 shows the ethanol and substrate concentrations during SSCF, while the data regarding by-product formation, xylose consumption and ethanol yield are summarized in Table 3. The KE6-12 xylose-fermenting yeast was first compared to baker’s yeast in SSCF of SPWS with batch addition of SWM. An ethanol concentration of 59.5 g/L (Figure 5A) was obtained with baker’s yeast, corresponding to a yield of 75% (Table 3), based on the total amount of glucose and xylose added to SSCF. As expected, the xylose decreased only slightly; 9% of the total amount added was converted, and almost all of it was reduced to xylitol. Although KE6-12 converted more xylose, 22% of the total, the ethanol production was slightly lower, 56.8 g/L ethanol corresponding to a 72% ethanol yield, based on both glucose and xylose, probably due to some xylitol (1.2 g/L) and significant glycerol (8.0 g/L) production. Xylitol excretion has been ascribed to an imbalance and insufficient NAD^+^ regeneration in XR for the XDH reaction [12,35]. NAD^+^ is produced by XR by the reduction of dihydroxyacetone phosphate to glycerol, which may explain the increase in glycerol production and reduction in xylitol production. Similar patterns have been observed previously in SSCF of wheat straw [36] and corn stover [37] using the parental strain TMB3400.

**Table 3:** Substrate, product and by-product concentrations (c) and yields (Y) obtained after 120 hours of SSCF with SWM addition

**Configuration**	**c**_**glu**_	**c**_**xyl**_	**c**_**EtoH**_	**c**_**glycerol**_	**Y**_**glycerol/glu**_	**c**_**xylitol**_	**Y**_**xylitol**_	**Xyl cons.**	**EtOH prod. rate**	**Y**_**EtOH/glu**_		**Y**_**EtOH/glu+xyl**_	
	g/L	g/L	g/L	g/L	%^a^	g/L	%^b^	%^c^	g/L/h^d^	%	g/g^a^	%	g/g^e^
A^SSF^ Batch	3.5	17.9	59.5	3.8	3.0	2.0	82	9	0.98	95	0.49	75	0.38
A^SSCF^ Batch	3.5	14.1	56.8	8.0	6.3	1.2	20	22	1.86	87	0.44	72	0.37
A^SSCF^ Fed-batch I	0.0	11.6	60.0	4.3	3.4	1.0	12	31	0.57	92	0.47	76	0.39
A^SSCF^ Fed batch II	0.0	14.5	58.8	3.5	2.7	1.0	17	21	0.86	90	0.46	74	0.38

The experiments were performed using the xylose-fermenting *S. cerevisiae* strain KE6-12 (Mean values are given for the reference SSF experiment using baker’s yeast: the standard deviation was less than 2%), glu: glucose, xyl: xylose, EtOH: ethanol.

^a^ Based on total amount of glucose added to SSF/SSCF.

^b^ Based on consumed xylose.

^c^ Based on total amount of xylose added to SSF/SSCF.

^d^ Calculated for the first 8 hours.

^e^ Based on total amount of glucose and xylose added to SSF/SSCF.

The significantly improved ethanol production rate using KE6-12 during the first 8 hours can be attributed to the general advantage of metabolic activity gained during cultivation (in comparison of dry yeast) and/or the cultivation of KE6-12 on lignocellulosic hydrolysate. It has previously been shown that yeast cultivation on lignocellulosic hydrolysate improves inhibitor tolerance considerably [24].

Fed-batch addition of SWM was performed in two ways: starting after 24 hours and continuing for 72 hours (24–96 hours), and starting after 48 hours and continuing for 48 hours (48–96 hours). Since the initial WIS concentration was considerably higher in this configuration (about 11 wt-%), than in batch-wise SSCF, the ethanol production rate was lower and glucose depletion was delayed. Hence, starting feeding after 24 hours, when the glucose was not fully depleted, led to glucose accumulation (Figure 5B), no improvement in xylose consumption was seen, and the ethanol yield was similar to that in the batch experiment. High glucose concentrations have been shown to reduce xylose utilization, as a result of the shared transport system for sugars in *S. cerevisiae*, which has a 200-fold greater affinity for glucose than xylose [12]. However, a low, but non-zero, glucose concentration should be maintained to prevent competitive inhibition of xylose transport by glucose [38]. Glucose and xylose co-fermentation has been thoroughly investigated in both SSCF [36,39-41] and separate hydrolysis and co-fermentation [13,42], and in all cases it was shown that maintaining a low glucose concentration during fermentation facilitated xylose uptake.

Better xylose utilization was observed when feeding started with a glucose feeding rate under 1 g/L/h after 48 hours, as the glucose had already been metabolized. The low glucose concentration after 48 hours made xylose uptake possible, although the xylose concentration was almost constant until the glucose had been depleted (see Figure 5C). Thirty-one percent of the xylose was consumed and the ethanol concentration was 60.0 g/L, the highest achieved in any of the SSCF experiments, corresponding to a fairly high ethanol yield of 76%, based on both xylose and glucose. Yields in the same range have been achieved by Olofsson et al. [40]; however, more efficient xylose consumption was obtained in that study by applying feeding of cellulosic enzymes. Furthermore, fed-batch addition of the lignocellulosic substrate is a reliable way of keeping the glucose concentration low [39,43], especially during the first 48 hours. It may thus be interesting to study fed-batch addition of lignocellulosic substrate in combination of SWM addition as the latter one would provide a source of glucose that can be fed at a low rate, ensuring a high xylose-to-glucose ratio, facilitating xylose utilization.

## Conclusions

The results of this study have shown that the addition of SWM leads not only to a significant increase in ethanol concentration, but also allows (for the configurations used in this study), the protein-rich solid residue from the wheat meal to be separated and used as animal feed. Ethanol concentrations of about 6% (w/v) were obtained, which will result in a significant reduction in the cost of downstream processing, compared with the SSF of the lignocellulosic substrate alone. Ethanol yields are also increased during SSF, probably due to the high proportion of easily fermentable sugar and additional sources of nutrients. Sugar losses must, however, be avoided to maximize overall yields. The studied configurations resulted in rather similar yields around 90%, which shows that these are rather robust processes.

Batch SSF with the addition of FWM suffered from inhibition mostly due to the high concentration of ethanol added together with the FWM. In the fed-batch configuration, a relatively high ethanol concentration, 51.2 g/L, corresponding to a 90% ethanol yield, was obtained despite some inhibition due to the slight accumulation of glucose during FWM feeding.

Finally, fed-batch addition of SWM was shown to increase the xylose uptake slightly in SSCF using the xylose-fermenting strain KE6-12. However, no increase in ethanol yield or concentration was obtained. Low-rate feeding of SWM after depletion of the glucose resulted in an ethanol concentration as high as 60.0 g/L, corresponding to an ethanol yield of 92%, which is in the same range as that achieved using baker’s yeast. Since a high glucose concentration during the first 48 hours prevented xylose utilization, the investigation of fed-batch addition of lignocellulosic substrate in the first period is of interest as a means of reducing inhibition and improving xylose uptake.

## Materials and methods

### Materials

The wheat straw used in this study was obtained from Johan Håkansson Lantbruksprodukter (Lunnarp, southern Sweden). The dry matter (DM) content was 91%, and the straw was stored at room temperature. The wheat meal was provided by Sileco (Laholm, Sweden). It had a particle size of 2.5-3 mm, a DM content of 89%, and contained 73.4% starch on a dry basis. It was stored in a plastic bucket at 5°C until used. The enzyme preparations, α-amylase (Termamyl SC; Novozymes A/S, Bagsvaerd, Denmark) was used for wheat meal liquefaction amyloglucosidase (Spirizyme Fuel; Novozymes A/S) for saccharification, and cellulases (Cellic Ctec2; Novozymes A/S) in SSF. Cellic Ctec2 had a filter paper activity of 98.7 FPU/mL. Dried baker’s yeast, *Saccharomyces cerevisiae* (Jästbolaget AB, Sweden) was used in fermentation of saccharified wheat meal and SSF. The yeast preparation had a dry cell content of 75.1%. In the SSCF experiments genetically modified xylose-fermenting yeast, KE6-12 (Taurus Energy AB, Sweden) was used.

### Substrate processing

#### Pretreatment of wheat straw

The composition of the wheat straw was determined according to the standard methods of the National Renewable Energy Laboratory (NREL) [44]. The straw consisted of 31.6% glucan, 22.0% xylan, 4.0% arabinan, 21.4% lignin, 1.5% starch, 12.6% extractives and 1.7% ash. The straw was cut in a knife mill (Retsch GmbH, Haan, Germany) and sieved to obtain particles in the range 2–10 mm. The particles were impregnated with dilute (0.2 wt-%) sulphuric acid solution (20 g liquid/g dry straw) for one hour. Excess liquid was removed after impregnation by pressing to a DM content of about 50 wt-% using a 5 L filter press (Fischer Maschinenfabrik GmbH, Germany). The pressed material was stored in plastic buckets at room temperature before steam pretreatment. Pretreatment was performed in a steam-pretreatment unit described previously [45], using saturated steam at 190°C for 10 min [46]. The SPWS slurry had a WIS content of 11.7 wt-% and was subsequently subjected to SSF. The compositions of the solid and the liquid fractions of the pretreated material are given in Table 4.

**Table 4:** Composition of the steam-pretreated wheat straw used in the experiments

**Solid fraction**	**% of WIS**
Glucan	59.3
Xylan	6.7
Galactan	BDL^*^
Arabinan	BDL^*^
Mannan	BDL^*^
Lignin	
Acid-insoluble	28.3
Acid-soluble	0.7
Lignin ash	3.1
**Liquid fraction**	**g/L**
Monomers	
Glucose	3.4
Xylose	24.5
Galactose	4.1
Arabinose	5.4
Mannose	1.1
Oligomers	
Glucose	5.0
Xylose	12.3
Galactose	1.3
Arabinose	0.3
Mannose	0.3
By-products	
Furfural	2.6
HMF	0.2
Acetic acid	2.4
Formic acid	0.4

^*^ Below detection limit.

#### Wheat meal liquefaction and saccharification

The liquefaction and subsequent saccharification of wheat meal was performed in a 3-L LABFORS fermentor (Infors HT, Switzerland). Batches weighing 2.5 kg were prepared by mixing hot tap water to wheat meal to a DM content of 35%. The pH was adjusted to 5.5 with 96% sulphuric acid. Termamyl SC was added to the wheat meal at 0.5 g enzyme/kg dry wheat meal, and liquefaction was performed at 85°C for 3 hours. After readjusting the pH to 4.2, Spirizyme Fuel was added at a ratio of 1 mL enzyme/kg DM wheat meal. Saccharification was carried out at 60°C for 24 hours. Saccharified wheat meal slurry was first centrifuged at 4000 rpm in 500-mL flasks and then at 4500 rpm in 50-mL centrifuge tubes for 10 min, to achieve better separation. The SWM, obtained after centrifugal separation from the solid residue, was subjected to fermentation alone or added to the SSF of SPWS. The glucose concentration of SWM was determined to be 318.6 g/L.

#### Fermentation of saccharified wheat meal prior to SSF

Fermentation of SWM to obtain FWM was performed in a 2-L fermentor (Infors AB, Bottmingen, Switzerland) with a final working volume of 1 L. Nutrients were dissolved separately in deionized water to final concentrations of 0.5 g/L (NH_4_)_2_HPO_4_, 0.025 g/L MgSO_4_ · 7H_2_O and 1 g/L yeast extract, sterilized and added to the bioreactor before inoculation. The medium was also supplemented with 0.125 mL Vitahop-LactoStab® (hop extract preparation, from BetaTec Hop products, Germany) before inoculation to prevent microbial infections. Fermentation was performed at pH 5, at 32°C. After 48 hours, fermentation was stopped, the broth was centrifuged, and the liquid fraction, denoted FWM was fed to the SSF. The experimental conditions, including the total amount of sugars added to fermentations are given in Table 1.

#### Simultaneous saccharification and (co-)fermentation

SSF and SSCF experiments were performed in 2-L fermentors (Infors AG, Bottmingen, Switzerland) with a working weight of 1 kg. The experimental conditions for SSF and SSCF are presented in Table 1.

The SSF/SSCF medium was supplemented with nutrients at concentrations of 0.5 g/L (NH_4_)_2_HPO_4_, 0.025 g/L MgSO_4_∙7H_2_O and 1.0 g/L yeast extract. The experiments were carried out at 35°C for 120 h, at pH 5.0 maintained with 10 (w/w)% NaOH. Cellic Ctec2 enzyme preparation was added to the fermentation vessels at the start of all SSF/SSCF runs at a loading of 20 FPU/g cellulose. The SSF or SSCF experiments were initiated by the addition of dried baker’s yeast or the xylose-fermenting yeast, KE6-12 (Albers *et al.*: Evolutionary engineering for development of improved xylose utilization capacity and inhibitor tolerance in an industrial Saccharomyces cerevisiae strain, manuscript in preparation), respectively, at a DW concentration of 5 g cells /L. Samples were withdrawn for analysis after 1, 3, 5, 8, 12, 24, 48, 96 and 120 hours.

#### Overall procedure for integrated ethanol production

The experimental configurations used to investigate ethanol production from wheat straw and wheat meal are illustrated in Figure 1.

In Configuration A, SWM (188 g) was mixed with SPWS. The filter cake of SWM was washed with water to remove the sugars and this washing liquid (146 g) was used to adjust the WIS of the SPWS/SWM mixture to 7.5%. The proportion of the materials (SPWS/SWM) subjected to SSF was based on equal amounts of each original raw material.

SSCF experiments were performed in batch and fed-batch mode in Configuration A to investigate the co-fermentation of glucose and xylose. When batch SSCF was performed, SWM mixed with washing liquid was added to the bioreactor at time 0, while fed-batch SSCF was performed by feeding the same mixture at two different feeding rates. Feeding was started after 24 or 48 hours at a glucose feed rate of 1.44 or 0.96 g/L/h, respectively, and continued until 96 hours.

In Configuration B, the WIS content in SSF was adjusted with SWM (220 g) to 8.8%, without any washing liquid. Reference experiments were performed with addition of water used for adjusting the WIS for 7.5% and 8.8%.

In Configuration C, the filter cake of SWM was washed prior to fermentation and the liquid from washing (WL) was used in fermentation to dilute SWM. The fermentation was started with an eight-hour batch phase, using 438 mL WL liquid (130.2 g/L glucose). A fed-batch phase was then performed using 552 mL SWM (318.6 g/L glucose), fed over a period of 24 h, at a constant feed-rate. After fermentation, the broth was separated from the yeast and the whole FWM (339 g) was added at the beginning of SSF to adjust the WIS concentration to 7.5 wt-%.

In Configuration D, the filter cake was not washed, and the water required for dilution was added directly to the fermentation step. The fermentation in this configuration was also started with an eight-hour batch phase, but using 157.2 mL SWM and 342.8 mL water (total 500 mL), resulting in an initial glucose concentration of 100 g/L. Following the batch phase, another 500 mL of a sugar-rich solution containing 405.2 mL SWM (glucose concentration 258.2 g/L) was fed to the fermentor over 24 hours. The broth of FWM (339 g) was then separated from the yeast and fed to SSF of SPWS between 24 and 96 hours.

#### Cultivation of the xylose-fermenting yeast

Genetically modified *Saccharomyces cerevisiae* KE6-12 cells (100 μL from a −80°C glycerol stock yeast culture) were added to 70 mL of an aqueous solution containing sugars (23.8 g/L glucose), salts (10.8 g/L (NH_4_)_2_SO_4_, 5.0 g/L KH_2_PO_4_, 1.1 g/L MgSO_4_·7H_2_O), 14.4 mL/L trace metal solution and 1.4 mL/L vitamin solution in a 300-mL Erlenmeyer flask. Trace metal and vitamin solutions were prepared as described by Taherzadeh et al. [47]. The pH was adjusted to pH 5 with 1 wt-% NaOH, the flask was sealed with a cotton plug, and incubated on a rotary shaker at 180 rpm for 24 h at 30°C.

Batch cultivation was performed in a 2-L LABFORS fermentor with a working volume of 0.5 L (Infors AG, Bottmingen, Switzerland). Cultivation was started by adding 70 mL inoculum to a medium containing 20.0 g/L glucose, 22.5 g/L (NH_4_)_2_SO_4_, 10.5 g/L KH_2_PO_4_, 2.2 g/L MgSO_4_·7H_2_O, 60.0 mL/L trace metal solution and 6.0 mL/L vitamin solution. Cultivation was carried out at pH 5, maintained with 10 wt-% NaOH, at 700 rpm with an aeration rate of 1.5 vvm. The dissolved oxygen concentration was measured continuously with an oxygen sensor. Fed-batch cultivation was started when the oxygen concentration increased rapidly, indicating that all the sugar and the ethanol had been consumed.

During the fed-batch phase, 921 mL pressed liquid of SPWS supplemented with glucose and salt solution to a total volume of 1 L was added to the fermentor. The glucose concentration in the liquid of SPWS was adjusted to 80.0 g/L and salts were added at concentrations of 11.3 g/L (NH_4_)_2_SO_4_, 5.3 g/L KH_2_PO_4_ and 1.1 g/L MgSO_4_·7H_2_O. The hydrolysate was fed to the fermentor at an increasing flow rate, to a maximum of 225 mL/h, for 24 hours. The culture broth was centrifuged at 4000 rpm for 10 min. The DM content of the harvested cells was determined before use in SSF.

### Sample characterization

The carbohydrate, soluble and insoluble lignin content in the solid fraction and total sugar content in the liquid fraction of SPWS were analysed according to NREL standard methods [44,48]. Samples taken from the liquid fraction of SPWS, from the fermentations, SSF and SSCF were centrifuged and the supernatants were filtered using 0.2 μm sterile filters (A Filter, Gothenburg, Sweden. The filtrates were stored in a freezer at −18°C. The samples were analysed using a high-performance liquid chromatograph equipped with a refractive index detector (both from Shimadzu, Kyoto, Japan). The sugar and xylitol concentrations were determined using an Aminex HPX-87P column (Bio-Rad Laboratories, Hercules, CA, USA) at 85°C with Millipore water as eluent at a flow rate of 0.5 mL/min. Ethanol, glycerol, lactic acid, acetate and degradation products such as HMF and furfural were separated on an Aminex HPX-87H column (Bio-Rad Laboratories) at 50°C. The eluent used was 5 mM H_2_SO_4_ at a flow rate of 0.5 mL/min.

### Yield calculations

Ethanol yields from the SSF experiments using baker’s yeast were calculated based on the total amount of glucose added, while in SSCF using KE6-12 the yield was based on the total amount of glucose and xylose added. The total (theoretical) amounts of glucose and xylose in the WIS fraction were calculated from the cellulose and xylan contents of the fibres multiplied by 1.11 and 1.13, respectively. The sugar contents in the liquid fractions were based on both monomer and oligomer sugars. Xylose consumption was calculated based on the total amount of xylan in the WIS, and xylose determined in the liquid fraction, including both monomers and oligomers. The ethanol yield was calculated based on the maximum ethanol yield of 0.51 g/g sugar.

## Abbreviations

DM: Dry matter; FWM: Fermented wheat meal; NREL: National renewable energy laboratory; SPWS: Steam-pretreated wheat straw; SSF: Simultaneous saccharification and fermentation; SSCF: Simultaneous saccharification and co-fermentation; SWM: Saccharified wheat meal; WIS: Water-insoluble solids.

## Competing interests

The authors declare that they have no competing interests.

## Authors’ contribution

BE, MG and GZ designed and coordinated the study. DH carried out the experiments. DH and BE analysed the results. BE prepared the manuscript. MG and GZ have reviewed the manuscript. All authors read and approved the final manuscript.

## References

[B1] Rosillo-Calle F Food versus fuel: toward a new paradigm - the need for a holistic approach ISRN Renewable Energy 2012 2012 954180

[B2] Sims REH Mabee W Saddler JN Taylor M An overview of second generation biofuel technologies Bioresour Technol 2010 101 1570 1580 10.1016/j.biortech.2009.11.046 19963372

[B3] Galbe M Sassner P Wingren A Zacchi G Olsson L Process engineering economics of bioethanol production Biofuels, Volume 108 2007 Leipzig: Springer Berlin Heidelberg 303 327 10.1007/10_2007_063 17541520

[B4] Wingren A Galbe M Zacchi G Techno-economic evaluation of producing ethanol from softwood: comparison of SSF and SHF and identification of bottlenecks Biotechnol Prog 2003 19 1109 1117 12892470 10.1021/bp0340180

[B5] Hoyer K Galbe M Zacchi G Production of fuel ethanol from softwood by simultaneous saccharification and fermentation at high dry matter content J Chem Technol Biotechnol 2009 84 570 577 10.1002/jctb.2082

[B6] Jorgensen H Vibe-Pedersen J Larsen J Felby C Liquefaction of lignocellulose at high-solids concentrations Biotechnol Bioeng 2007 96 862 870 10.1002/bit.21115 16865734

[B7] Erdei B Barta Z Sipos B Réczey K Galbe M Zacchi G Ethanol production from mixtures of wheat straw and wheat meal Biotechnol Biofuels 2010 3 16 10.1186/1754-6834-3-16 20598120 PMC2912878

[B8] Tang Y Zhao D Cristhian C Jiang J Simultaneous saccharification and cofermentation of lignocellulosic residues from commercial furfural production and corn kernels using different nutrient media Biotechnol Biofuels 2011 4 22 10.1186/1754-6834-4-22 21801455 PMC3161845

[B9] Hahn-Hägerdal B Wahlbom CF Gárdonyi M van Zyl WH Cordero Otero RR Jönsson JJ Metabolic engineering of *Saccharomyces cerevisiae* for xylose utilization Adv Biochem Eng Biotechnol 2001 73 53 84 11816812 10.1007/3-540-45300-8_4

[B10] Wahlbom CF van Zyl WH Jonsson LJ Hahn-Hägerdal B Otero RRC Generation of the improved recombinant xylose-utilizing *Saccharomyces cerevisiae* TMB 3400 by random mutagenesis and physiological comparison with *Pichia stipitis* CBS 6054 FEMS Yeast Res 2003 3 319 326 10.1016/S1567-1356(02)00206-4 12689639

[B11] Eliasson A Christensson C Wahlbom CF Hahn-Hägerdal B Anaerobic xylose fermentation by recombinant *Saccharomyces cerevisiae* carrying XYL1, XYL2, and XKS1 in mineral medium chemostat cultures Appl Environ Microbiol 2000 66 3381 3386 10.1128/AEM.66.8.3381-3386.2000 10919795 PMC92159

[B12] Kötter P Ciriacy M Xylose fermentation by *Saccharomyces cerevisiae* Appl Microbiol Biotechnol 1993 38 776 783 10.1007/BF00167144

[B13] Erdei B Frankó B Galbe M Zacchi G Glucose and xylose co-fermentation of pretreated wheat straw using mutants of S. cerevisiae TMB3400 J Biotechnol 2013 164 50 58 10.1016/j.jbiotec.2012.12.003 23262129

[B14] Koppram R Nielsen F Albers E Lambert A Wannstrom S Welin L Zacchi G Olsson L Simultaneous saccharification and co-fermentation for bioethanol production using corncobs at lab, PDU and demo scales Biotechnol Biofuels 2013 6 2 10.1186/1754-6834-6-2 23311728 PMC3598390

[B15] Hallsworth JE Ethanol-induced water stress in yeast J Ferment Bioeng 1998 85 125 137 10.1016/S0922-338X(97)86756-6

[B16] Ingledew WM Jacques KA, Lyons TP, Kelsall DR Alcohol production by Saccharomyces cerevisiae: a yeast primer The alcohol textbook 1999 Nottingham, United Kingdom: Nottingham University Press 49 87

[B17] Kenyon CP Prior BA van Vuuren HJJ Water relations of ethanol fermentation by Saccharomyces cerevisiae: Glycerol production under solute stress Enzyme Microb Technol 1986 8 461 464 10.1016/0141-0229(86)90047-5

[B18] Elliott D Orth R Gao J Werpy T Eakin D Schmidt A Neuenschwander G Murry J Flagg A Lahman L Biorefinery concept development based on wheat flour milling 2002 Richland, WA (US: Pacific Northwest National Laboratory (PNNL)

[B19] Sanchez B Bautista J Effects of furfural and 5-hydroxymethylfurfural on the fermentation of *Saccharomyces cerevisiae* and biomass production from *Candida guilliermondii* Enzyme Microb Technol 1988 10 315 318 10.1016/0141-0229(88)90135-4

[B20] Taherzadeh MJ Gustafsson L Niklasson C Liden G Physiological effects of 5-hydroxymethylfurfural on *Saccharomyces cerevisiae* Appl Microbiol Biotechnol 2000 53 701 708 10.1007/s002530000328 10919330

[B21] Larsson S Palmqvist E Hahn-Hägerdal B Tengborg C Stenberg K Zacchi G Nilvebrant NO The generation of fermentation inhibitors during dilute acid hydrolysis of softwood Enzyme Microb Technol 1999 24 151 159 10.1016/S0141-0229(98)00101-X

[B22] Tengborg C Galbe M Zacchi G Reduced inhibition of enzymatic hydrolysis of steam-pretreated softwood Enzyme Microb Technol 2001 28 835 844 10.1016/S0141-0229(01)00342-8 11397466

[B23] Ohgren K Rudolf A Galbe M Zacchi G Fuel ethanol production from steam-pretreated corn stover using SSF at higher dry matter content Biomass Bioenerg 2006 30 863 869 10.1016/j.biombioe.2006.02.002

[B24] Alkasrawi M Rudolf A Lidén G Zacchi G Influence of strain and cultivation procedure on the performance of simultaneous saccharification and fermentation of steam pretreated spruce Enzyme Microb Technol 2006 38 279 286 10.1016/j.enzmictec.2005.08.024

[B25] Tomás-Pejó E Negro MJ Sáez F Ballesteros M Effect of nutrient addition on preinoculum growth of S. cerevisiae for application in SSF processes Biomass Bioenerg 2012 45 168 174

[B26] Albertyn J Hohmann S Thevelein JM Prior BA GPD1, which encodes glycerol-3-phosphate dehydrogenase, is essential for growth under osmotic stress in Saccharomyces cerevisiae, and its expression is regulated by the high-osmolarity glycerol response pathway Mol Cell Biol 1994 14 4135 4144 8196651 10.1128/mcb.14.6.4135-4144.1994 PMC358779

[B27] Puligundla P Smogrovicova D Obulam VSR Ko S Very high gravity (VHG) ethanolic brewing and fermentation: a research update J Ind Microbiol Biotechnol 2011 38 1133 1144 10.1007/s10295-011-0999-3 21695540

[B28] Palmqvist B Lidén G Torque measurements reveal large process differences between materials during high solid enzymatic hydrolysis of pretreated lignocellulose Biotechnol Biofuels 2012 5 57 10.1186/1754-6834-5-57 22867035 PMC3502536

[B29] Szulczyk KR McCarl BA Cornforth G Market penetration of ethanol Renewable Sustainable Energy Rev 2010 14 394 403 10.1016/j.rser.2009.07.007

[B30] Kim Y Mosier NS Hendrickson R Ezeji T Blaschek H Dien B Cotta M Dale B Ladisch MR Composition of corn dry-grind ethanol by-products: DDGS, wet cake, and thin stillage Bioresour Technol 2008 99 5165 5176 10.1016/j.biortech.2007.09.028 17988859

[B31] Qiu H Sun L Huang J Rozelle S Liquid biofuels in China: current status, government policies, and future opportunities and challenges Renewable Sustainable Energy Rev 2012 16 3095 3104 10.1016/j.rser.2012.02.036

[B32] Galbe M Zacchi G Pretreatment: the key to efficient utilization of lignocellulosic materials Biomass Bioenerg 2012 46 70 78

[B33] Piper PW The heat shock and ethanol stress responses of yeast exhibit extensive similarity and functional overlap FEMS Microbiol Lett 1995 134 121 127 10.1111/j.1574-6968.1995.tb07925.x 8586257

[B34] Casey GP Ingledew WMM Ethanol tolerance in yeasts CRC Crit Rev Microbiol 1986 13 219 280 10.3109/10408418609108739 3533426

[B35] Jeffries T Jin Y Metabolic engineering for improved fermentation of pentoses by yeasts Appl Microbiol Biotechnol 2004 63 495 509 10.1007/s00253-003-1450-0 14595523

[B36] Olofsson K Rudolf A Lidén G Designing simultaneous saccharification and fermentation for improved xylose conversion by a recombinant strain of Saccharomyces cerevisiae J Biotechnol 2008 134 112 120 10.1016/j.jbiotec.2008.01.004 18294716

[B37] Ohgren K Bengtsson O Gorwa-Grauslund M Galbe M Hahn-Hägerdal B Zacchi G Simultaneous saccharification and co-fermentation of glucose and xylose in steam-pretreated corn stover at high fiber content with Saccharomyces cerevisiae TMB3400 J Biotechnol 2006 126 488 498 10.1016/j.jbiotec.2006.05.001 16828190

[B38] Meinander NQ Boels I Hahn-Hägerdal B Fermentation of xylose/glucose mixtures by metabolically engineered *Saccharomyces cerevisiae* strains expressing XYL1 and XYL2 from *Pichia stipitis* with and without overexpression of TAL1 Bioresour Technol 1999 68 79 87 10.1016/S0960-8524(98)00085-6

[B39] Bertilsson M Olofsson K Lidén G Prefermentation improves xylose utilization in simultaneous saccharification and co-fermentation of pretreated spruce Biotechnol Biofuels 2009 2 1 10 10.1186/1754-6834-2-1 19356227 PMC2671495

[B40] Olofsson K Wiman M Lidén G Controlled feeding of cellulases improves conversion of xylose in simultaneous saccharification and co-fermentation for bioethanol production J Biotechnol 2010 145 168 175 10.1016/j.jbiotec.2009.11.001 19900494

[B41] Rudolf A Baudel H Zacchi G Hahn-Hägerdal B Lidén G Simultaneous saccharification and fermentation of steam-pretreated bagasse using *Saccharomyces cerevisiae* TMB3400 and *Pichia stipitis* CBS6054 Biotechnol Bioeng 2008 99 783 790 10.1002/bit.21636 17787015

[B42] Erdei B Frankó B Galbe M Zacchi G Separate hydrolysis and co-fermentation for improved xylose utilization in integrated ethanol production from wheat meal and wheat straw Biotechnol Biofuels 2012 5 12 10.1186/1754-6834-5-12 22410131 PMC3350417

[B43] Olofsson K Palmqvist B Lidén G Improving simultaneous saccharification and co-fermentation of pretreated wheat straw using both enzyme and substrate feeding Biotechnol Biofuels 2010 3 17 20678195 10.1186/1754-6834-3-17 PMC2923126

[B44] Sluiter A Hames B Ruiz R Scarlata C Sluiter J Templeton D Crocker D Determination of structural carbohydrates and lignin in biomass Golden 2008 National Renewable Energy Laboratory: Colorado

[B45] Palmqvist E Hahn-Hägerdal B Galbe M Larsson S Stenberg K Szengyel Z Tengborg C Zacchi G Design and operation of a bench-scale process development unit for the production of ethanol from lignocellulosics Bioresour Technol 1996 58 171 179 10.1016/S0960-8524(96)00096-X

[B46] Linde M Jakobsson EL Galbe M Zacchi G Steam pretreatment of dilute H_2_SO_4_ -impregnated wheat straw and SSF with low yeast and enzyme loadings for bioethanol production Biomass Bioenerg 2008 32 326 332 10.1016/j.biombioe.2007.09.013

[B47] Taherzadeh MJ Lidén G Gustafsson L Niklasson C The effects of pantothenate deficiency and acetate addition on anaerobic batch fermentation of glucose by *Saccharomyces cerevisiae* Appl Microbiol Biotechnol 1996 46 176 182 10.1007/s002530050801 8987648

[B48] Sluiter A Hames B Ruiz R Scarlata C Sluiter J Templeton D Determination of sugars, byproducts, and degradation products in liquid fraction process samples 2006 National Renewable Energy Laboratory: Golden, Colorado

